# Fabrication of high-resolution nanostructures of complex geometry by the single-spot nanolithography method

**DOI:** 10.3762/bjnano.6.101

**Published:** 2015-04-17

**Authors:** Alexander Samardak, Margarita Anisimova, Aleksei Samardak, Alexey Ognev

**Affiliations:** 1 Laboratory of Thin Film Technologies, School of Natural Sciences, Far Eastern Federal University, 8 Sukhanova St., Vladivostok 690950, Russia

**Keywords:** electron-beam lithography, exposure dose, high-resolution lithography, nanomagnets, nanostructure, overexposure, PMMA, polymer, resist carbonization

## Abstract

The paper presents a method for the high-resolution production of polymer nanopatterns with controllable geometrical parameters by means of a single-spot electron-beam lithography technique. The essence of the method entails the overexposure of a positive-tone resist, spin-coated onto a substrate where nanoscale spots are exposed to an electron beam with a dose greater than 0.1 pC per dot. A single-spot enables the fabrication of a nanoring, while a chain of spots placed at distance of 5–30 nm from each other allows the production of a polymer pattern of complex geometry of sub-10 nm resolution. We demonstrate that in addition to the naturally oxidized silicon substrates, gold-coated substrates can also successfully be used for the single-spot nanopattering technique. An explanation of the results related to the resist overexposure was demonstrated using Monte Carlo simulations. Our nanofabrication method significantly accelerates (up to 10 times) the fabrication rate as compared to conventional lithography on positive-tone resist. This technique can be potentially employed in the electronics industry for the production of nanoprinted lithography molds, etching masks, nanoelectronics, nanophotonics, NEMS and MEMS devices.

## Introduction

The continuous growth of the nanotechnology and microelectronic industries requires the development of new approaches and methods for the formation of nanoscale structures of desired geometry with a spatial resolution of less than 100 nm. One of the most advanced and in-demand technologies is mask-free or direct-write lithography based on the interaction of an electron beam with a polymer resist [1].

Under normal conditions, electron-beam lithography (EBL) enables the production of polymer patterns with an ≈20–30 nm spatial resolution (e.g., line width or dot diameter) of smallest elements [2]. Under certain conditions (exposure at an acceleration voltage >50 kV, development at low temperatures, using a dose correction mechanism, etc.), a resolution of 5 nm for single lines and dots can be achieved [3–5]. Since this is the absolute best-case resolution, it is almost impossible to obtain, for example, rectangular or triangular nanoobjects with sharp edges and low roughness. For drawing arrays consisting of more than 10,000 nanoscale elements, the time required for a template pattering can be estimated to be on the order of hours or even tens of hours. Often, during such a long exposure, a pattern generator will produce an error due to a buffer overflow because of the large data exchange. Moreover, the main drawback of EBL is the exposure speed (usually 10^7^ pixels per second) [6] and the expense involved in the mass production process. One possible solution to these mentioned issues is to use the dual-tone property of polymer resists, which depends on the exposure dose and enables the fast and inexpensive fabrication of high-resolution nanopatterns of complex shape.

The effect of high-dose electron irradiation on the chemical properties of polymers (resulting in hardening or overexposure) was observed in different types of positive resists: DQN [7], AZ-PF514 [8], and PMMA [9–10]. Poly(methyl methacrylate) (PMMA) is the most prevalent EBL resist employed due to its high-resolution capability, good lift-off performance, stability, excellent adhesion to substrates and compatibility with the other processing steps. PMMA consists of long polymer chains that are broken into smaller, more soluble fragments by scission with the electron beam. Depending on the exposure dose, PMMA can act as a positive or negative resist [9–10]. PMMA’s dual-tone resist capability opens up a simple and inexpensive route to produce a range of polymer nanostructures using direct overexposure of the electron beam [11–15].

In this paper we report on the low acceleration voltage (<30 kV) fabrication of polymer nanopatterns of different geometry consisting of both the individual elements and arrays of nanostructures with sub-20 nm resolution on semiconductor and metallic surfaces.

## Results and Discussion

### Single-spot pattering of polymeric micro- and nano-rings on silicon substrates

In the first experiments, the naturally oxidized, monocrystalline silicon substrates were spin-coated with PMMA 950k A2 positive-tone resist to a thickness of 150 nm. The acceleration voltage of the electron beam was 10 kV. The magnitude of the acceleration voltage determines the energy of the electron beam, and hence the penetration depth into the resist and substrate. The exposure dose refers to the number of inserted electrons in the primary electron beam. In the beginning, it was empirically found that at single spot doses less than 0.2 pC, the PMMA resist behaves as positive-tone resist. The high solubility of the positive-tone resist in the exposed areas was observed after development in a 1:3 4-methyl-2-pentanone (MIBK)/IPA solution for 30 s under normal conditions. Soaking the resist in acetone for 30 s leads to the complete removal of any remaining resist. At single-spot doses in the range 0.2–100 pC, the exposed and developed samples showed the dual-tone resist behavior.

**Figure 1:**
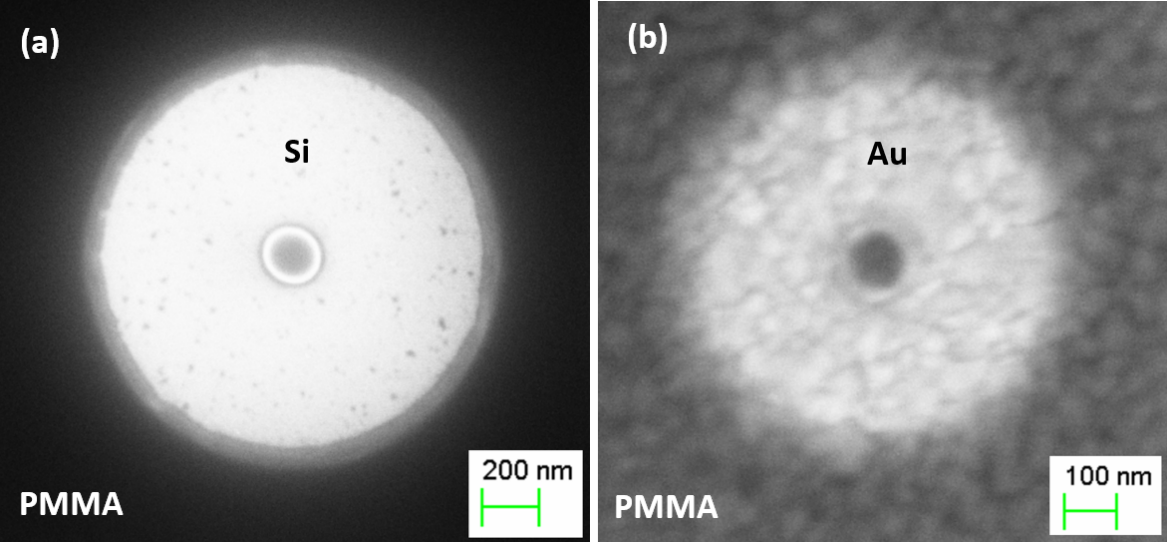
SEM images demonstrating the effect of substrate on the single-spot overexposure of a ring at a dose of 1 pC on 150 nm thick PMMA at an acceleration voltage of 10 kV: (a) single-crystal Si substrate; (b) Si substrate coated with a 200 nm Au film. Note the difference in scale between the two images.

As seen in Figure 1a, the pattern prepared with a single electron-beam spot is ring-shaped: the resist remains in the center surrounded by a clean substrate area. The main reason for the formation of the ring is the overexposure of the resist at the irradiation point followed by its carbonization [11,13]. Thus, the overexposed, positive resist acts as a negative resist. The distribution of electrons in a resist due to forward and back scattering is Gaussian in nature. This means that only the central part of the exposed area receives a dose with a value above the threshold for the carbonization process. This fact is confirmed by the high chemical resistance of the ring core to developer and solvents such as acetone and EBR remover. The surrounding resist receives a significantly lower dose and remains as a positive-tone resist that can be easily removed with developer or acetone. As discussed below, the diameter of the core and the outer ring size depend not only on the exposure dose, but also on the thickness of the resist, acceleration voltage of the electron beam, and the atomic number, roughness and crystal structure of substrate materials.

The substrate material has an important role in the formation of polymer patterns. Carbonized ring cores, formed on Si and Au surfaces (Figure 1a,b), have the same round shape, while the diameter and the quality of core and outer edges vary significantly. In the case of a monocrystalline Si substrate, the ring has a sharp edge with a small resist undercut (Figure 1a). As seen in Figure 1b, exposure of the resist on the polycrystalline Au film leads to a deep undercut and rough edges after development.

**Figure 2:**
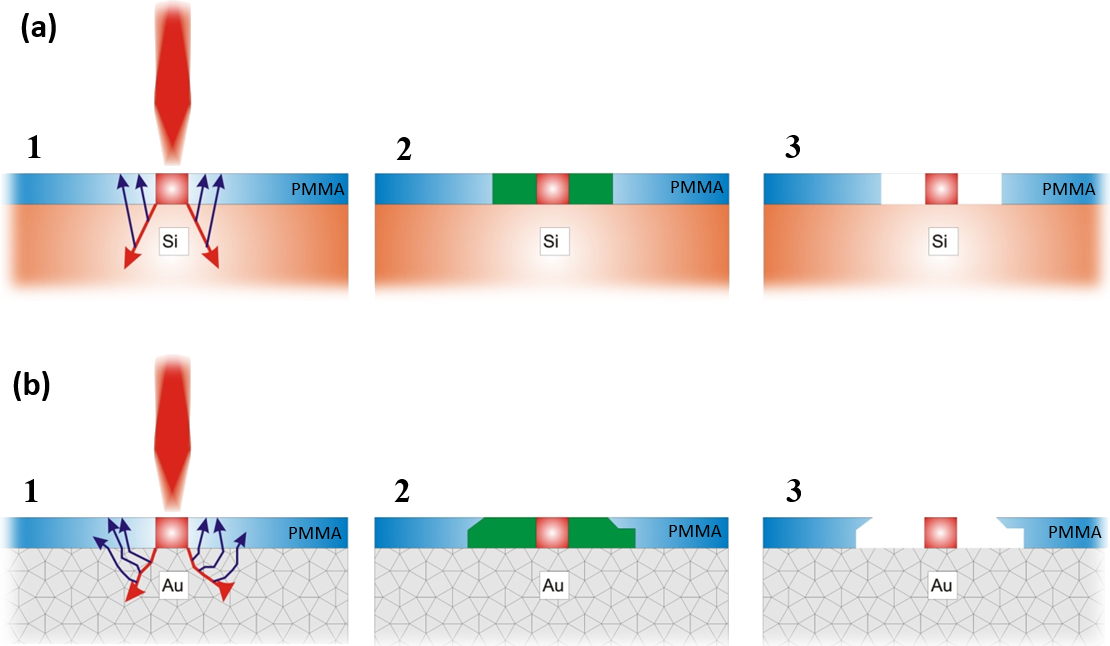
Single-spot overexposure of a ring dependening on the substrate type: (a) a single-crystal Si substrate and (b) a Si substrate coated with a 200 nm Au film. 1: an interaction of forward- and back-scattered electrons with positive resist; the overexposed area shown in red. 2: a normal exposure of resist areas highlighted in green. 3: the expected pattern profile after development.

The physics behind the single-spot overexposure and development process is schematically illustrated in Figure 2. The main impact area of the incident electron beam falls on the small central area, followed by the forward scattering of electrons in resist, which makes the beam diameter larger and leads to resist overexposure at high doses. The surrounding area of the resist is exposed due to backscattered and secondary electrons after large angle collisions in a substrate. These scattered electrons re-emerge into the resist at some distance from the point at which they left it. Forward and backscattered electrons have a distinct energy density distribution in the substrate [13], which defines the pattern profile after development.

**Figure 3:**
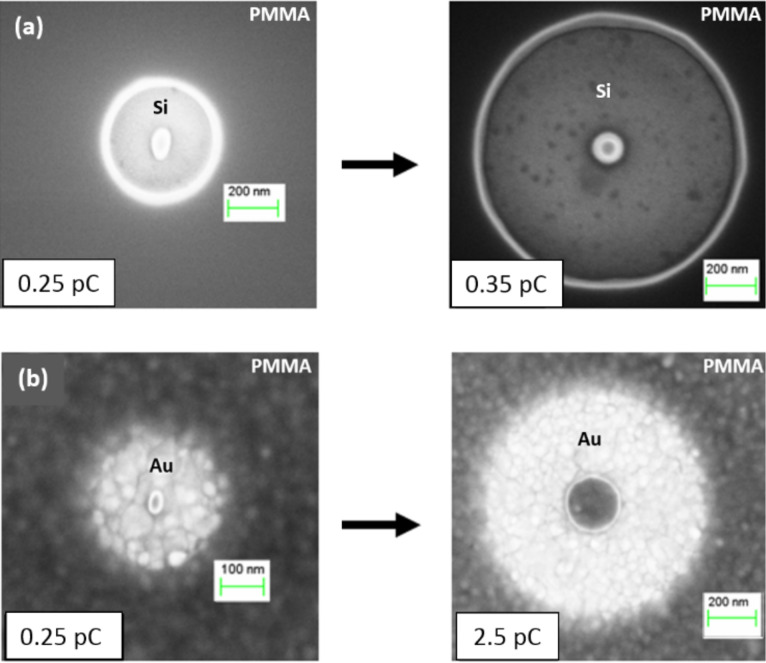
Effect of the exposure dose and substrate material on the single-spot pattern formation: (a) the ring prepared on a single-crystal Si substrate at a dose of 0.25 pC (left) and 0.35 pC (right); (b) the ring patterned on a polycrystalline Au film at a dose of 0.25 pC (left) and 2.5 pC (right).

Since elastic collisions of primary electrons in a substrate depend on the atomic number, *Z*, of the substrate material, the distribution of backscattered electrons in a Au film (*Z* = 79) will be significantly different than in Si (*Z* = 14) (Figure 3). As shown in [16], the backscattering coefficient, which defines a fraction of primary electrons that are backscattered in a substrate, varies significantly with *Z*. As Si is lighter than Au, the maximum of the backscattered electron emission is very broad and corresponds to the normalized energy, *W* = *E*/*E*_0_ = 0.5, where *E* and *E*_0_ are the energy of backscattered and primary electrons, respectively. For Au, *W* = 0.95–0.98. As a result, in the case of the Au substrate, the rings have a much smaller outer diameter compared to the Si substrate (Figure 3). Even at a dose of 2.5 pC, the ring on the Au substrate is still smaller than that patterned onto the Si substrate at a dose 0.35 pC.

**Figure 4:**
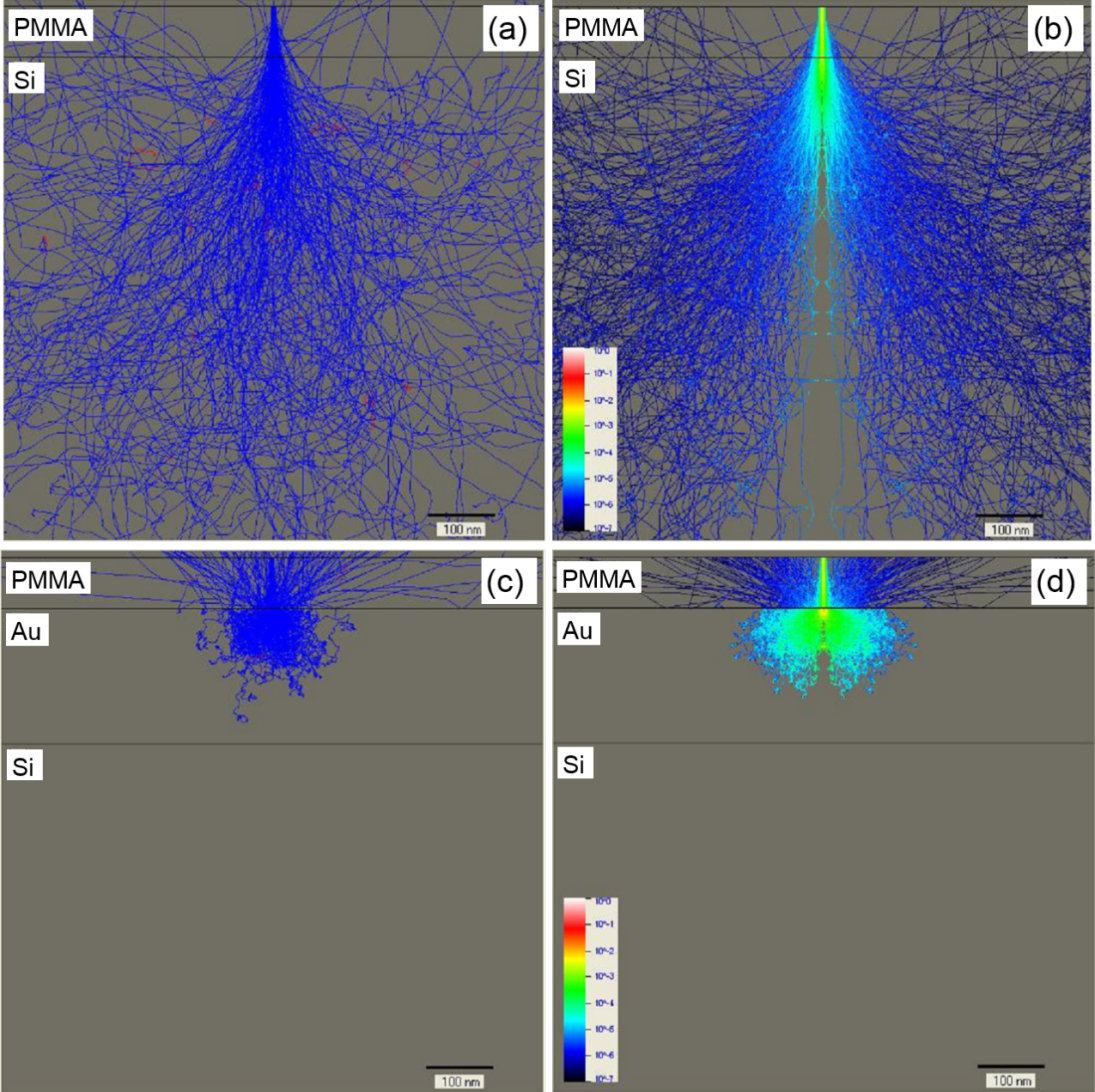
(a,c) Monte Carlo simulation of 250 electron scattering trajectories at 10 keV incident energy in a 75 nm thick PMMA layer on bulk Si and on 200 nm Au-coated substrates, respectively. (b,d) Electron energy distribution at 10 keV incident energy in a 75 nm thick PMMA layer on bulk Si and 200 nm Au-coated substrates, respectively. Trajectories that leave the sample represent backscattered electrons.

Our experimental findings are supported by Monte Carlo simulations performed with NanoPECS software [17]. In the case of the Si substrate, most of the electrons penetrate the resist via forward scattering at small angles, which broadens the primary beam size (Figure 4a). The energy distribution of electrons in Figure 4b shows that the central part of the resist is overexposed. Afterwards, the electrons enter into the Si substrate, where they collide with the nuclei of the atoms. If an electron has kinetic energy above the interaction treshold, three random scenarios are possible: small-angle forward scattering, wide-angle back scattering or secondary electron emission. Backscattered electrons are the main cause of the subsequent exposure of resist areas a few hundred nanometers away from the incident beam point (Figure 4a,b).

For the Au substrate (Figure 4c,d), significant wide-angle scattering occurs near the surface for a smaller interaction volume because of the heavy Au nucleus. Hence, a large fraction of incident electrons are backscattered to the resist not far away from the point where they penetrate into the Au film. As result, the outer edge of a ring patterned on a Au film has a smaller diameter than that on a Si substrate and becomes blurred. In contrast, for a single-crystal Si substrate, the electron scattering occurs with less variation (Figure 4a,b), which increases the sharpness of the core and outer edge (Figure 3).

**Figure 5:**
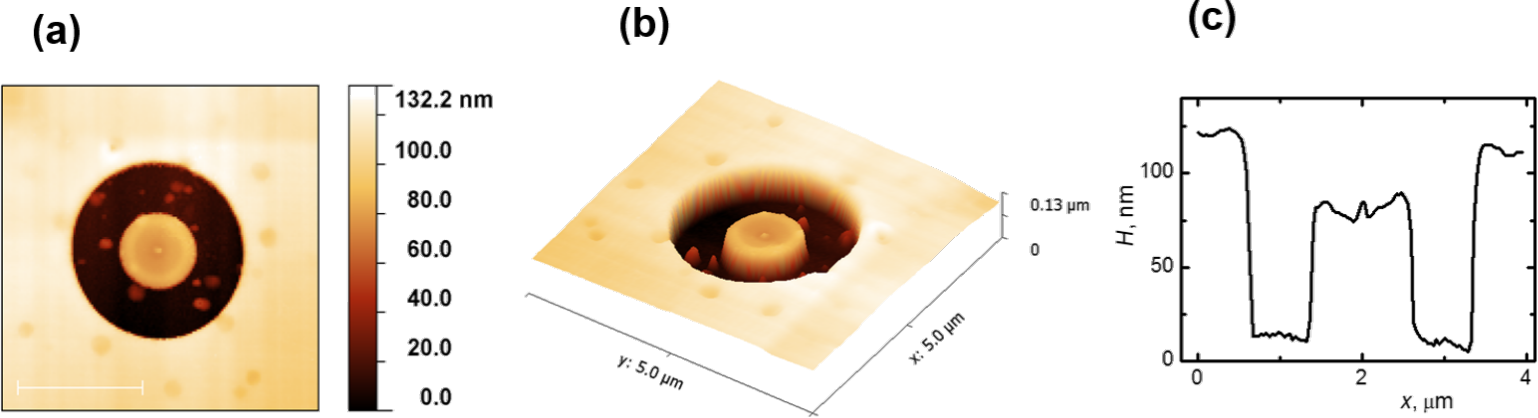
AFM images of the ring, patterned on the silicon substrate coated by a 120 nm thick PMMA A2 resist: (a) top view; (b) 3D view; (c) ring profile.

The spatial structure and profile of a ring measured by atomic force microscopy are shown in Figure 5. As seen in Figure 5c, after development, the height of the central pillar of the ring was reduced relative to the surrounding resist. It exhibits the characteristic centered peak, indicating the place where the incident electron beam interacted with resist. One explanation for this reduced height effect is the gradual dissipation of primary electrons that penetrate the resist. The single spot with Gaussian shape matching the electron beam diameter has the size of 2 nm on the resist surface. As it passes through the PMMA layer, the electron beam becomes broader because of the forward scattering arising from electron–electron interactions, and it carbonizes more resist volume being closer to the substrate. Thus, a trajectory distribution of electrons produced by Monte Carlo simulations shows that the upper third part of the pillar is very narrow and can be easily removed by developer (Figure 4).

**Figure 6:**
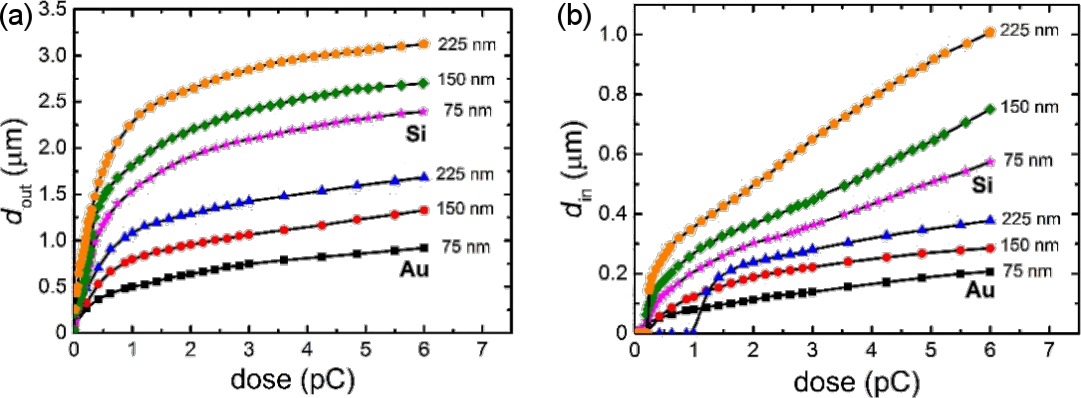
Dependence of *d*_out_ (a) and *d*_in_ (b) on the exposure dose at an acceleration voltage of 10 kV for different thicknesses of PMMA (from 75 to 225 nm) for Si and Au substrates.

Figure 6 shows the experimental dependence of the outer diameter *d*_out_ and the core diameter *d*_in_ on the radiation dose for silicon and gold-coated substrates with respect to resist thickness. Increasing the dose leads to an increase in both diameters. Thus, rings with much larger diameters can be patterned on the silicon substrate at a lower dose. As Au is a material with a much higher *Z* as compared to Si, the elastic scattering of the electrons in the Au substrate plays a key role. It leads to a large decrease of the interaction value, causing much smaller *d*_out_ and *d*_in_ values for the rings patterned using the same parameters on a Si substrate [16]. Moreover, it was experimentally found that the thickness of the resist also significantly contributes to the formation of the nanostructures. Thus, with increasing resist thickness, the values of *d*_out_ and *d*_in_ increase for both substrates. Primarily, this is caused by the increased broadening of the incident beam due to inelastic forward scattering of primary electrons in thicker resists, leading to a larger interaction volume in the substrate below. As shown in Figure 6b, the ring core begins to appear in the thinnest of the resists at a dose of 0.2 pC for both Si and Au substrates. For the thickest of the resists, this occurs at doses of 0.25 and 1.2 pC for Si and Au substrates, respectively.

As seen in Figure 6, the use of a polycrystalline conductive substrate, such as bulk Si coated with a thin Au film, gives the advantage of fabrication of rings with an outer diameter of less than 1 µm due to the saturation effect caused by a partial absorption of electrons in the metal film. However, for a single-crystal semiconductor substrate, such as Si, patterned rings of high resolution covering a wide range of possible diameters is feasible. The intentional change in the film thickness and crystallinity (grain size, lattice type) of the metal substrate enables the reflection and absorption of electrons to be manipulated and can be used for precise tuning of the parameters of the pattern.

### Patterning of complex nanostructures of various geometries

**Figure 7:**
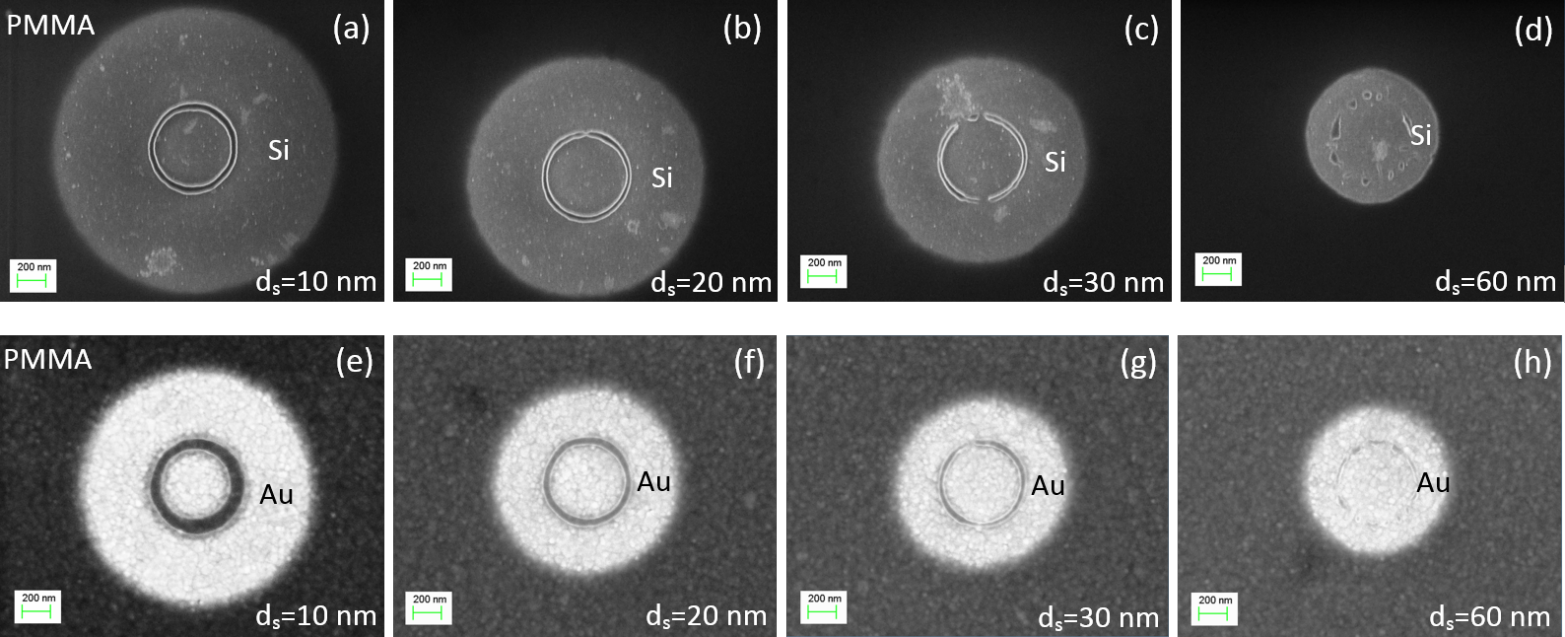
SEM images demonstrating the effect of changing the *d_s_* between the electron beam spots with a dose of 0.1 pC on the formation of circles on Si (a–d) and Au (e–h) substrates coated with PMMA of 75 nm thickness at an acceleration voltage of 10 kV.

For the fabrication of polymer nanostructure patterns of complex geometry, the Si substrate was spin-coated with a PMMA A2 resist. Before exposure, a special digital nanostructure pattern was prepared. Such a pattern consisted of a number of single spots with designated doses of 0.1 pC or higher. The dose 0.1 pC corresponds to the single-line dose of 10^5^ µC/cm, which is 100 times higher than the positive-tone regime in conventional lithography. The spots were arranged in the form of the desired nanostructure. It was experimentally discovered that the distance between neighboring spots, *d*_s_, must be ≤30 nm. At these values the carbonized cores coalesce during overexposure. After development, the surrounding area, which still behaves as a positive resist, is removed and the remaining carbonized resist forms a nanostructure in accordance with the digital pattern (Figure 7). In the case of the Si substrate, the smaller the *d*_s_, the higher the resolution, as demonstrated in Figure 7a–c. For the Au-coated substrate, larger distances up to *d*_s_ = 30 nm lead to higher resolution, as shown in Figure 7e–g. Since the electron beam diameter is approximately 2 nm, there is no advantage in designing a *d*_s_ less than 4 nm. For *d*_s_ > 30 nm, the links between overexposed areas are broken, leading to formation of separated islands of carbonized resist for both substrates (Figure 7d,h).

**Figure 8:**
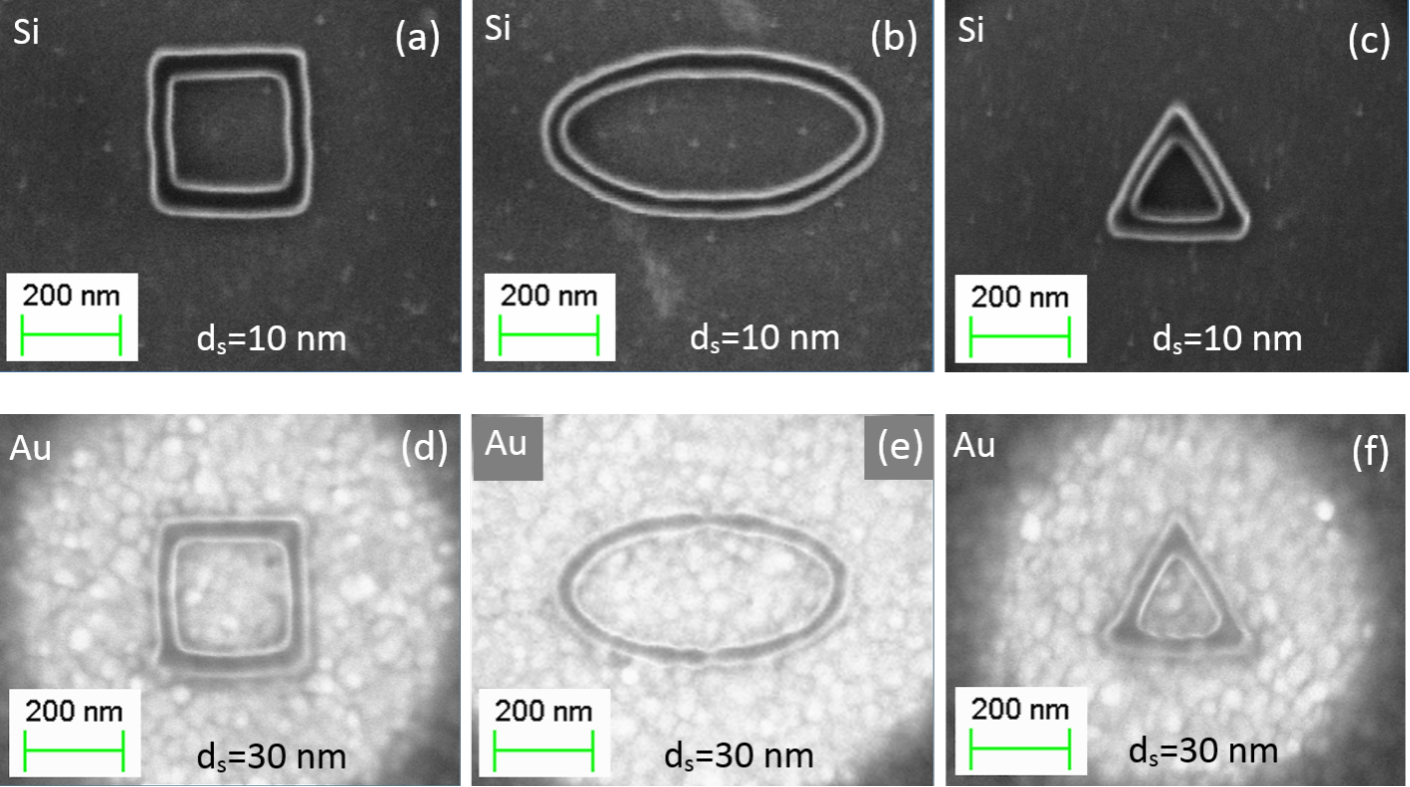
Polymer nanostructures fabricated on a PMMA A2 resist of 75 nm thickness at an acceleration voltage of 10 kV and exposure dose of 0.1 pC with *d*_s_ = 10 nm and 30 nm for Si (a–c) and Au (d–f) substrates, respectively. Patterned (a,d) square, (b,e) oval, and (c,f) triangle shapes possible using the methods described herein.

A few examples of nanostructures with a line width ranging from 10–30 nm patterned on Si and Au-coated substrates are shown in Figure 8. Patterns having the same resolution can be produced on monocrystalline Si and polycrystalline Au substrates, but at different values of *d*_s_. In the case of the Au substrate, *d*_s_ is three times larger than for Si. This enables a much faster fabrication of nanopatterns of the same dimension and quality on polycrystalline conductive substrates. The main reason for the highest resolution at *d*_s_ = 30 nm on the Au substrate is that the electron backscattering coefficient for bulk Au is about three times larger than for bulk Si at an acceleration voltage of 10 kV [18].

**Figure 9:**
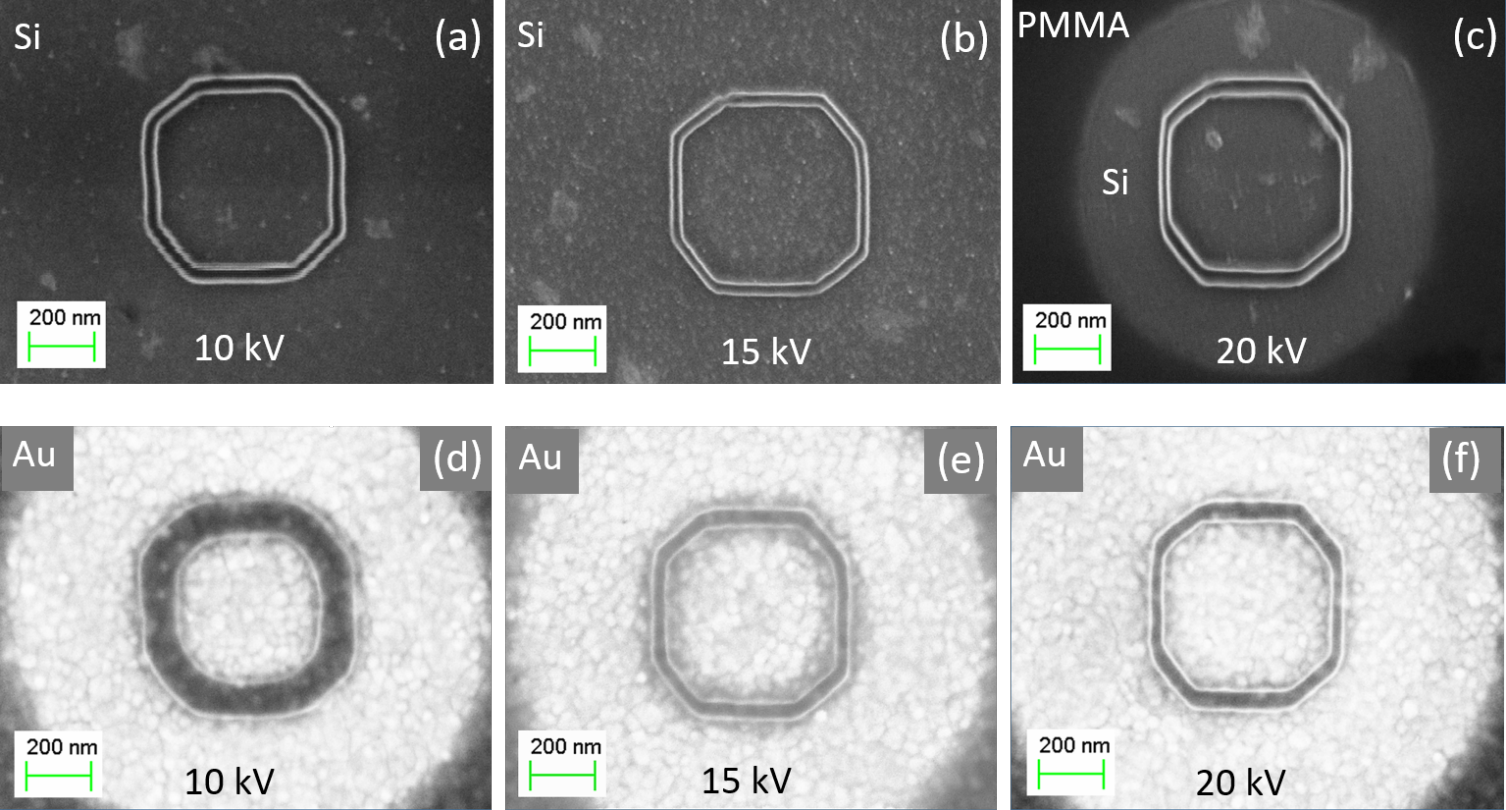
Effect of the acceleration voltage on the formation of polygons on Si (a–c) and Au (d–f) substrates coated with PMMA of 75 nm thickness and *d*_s_ = 10 nm.

The electron beam energy plays a very important role in the fabrication process because of its strong effect on the electron penetration depth and the number of inelastic collisions occurring in the resist. On the one hand, with an increase of the electron energy, the penetration depth increases, giving a narrow energy density distribution of primary electrons in the resist. On the other hand, at higher voltages, the number of polymer chain scissions per electron decreases. In Figure 9 the effect of the acceleration voltage on a nanopattern is represented. As seen in Figure 9a–c for a Si substrate, an increase in the acceleration voltage from 10 to 20 kV does not significantly change the resolution of the polygon. However, for the Au-coated substrate a completely different situation is observed (Figure 9d–f): with the increase of the acceleration voltage, the resolution/resist contrast is drastically improved.

**Figure 10:**
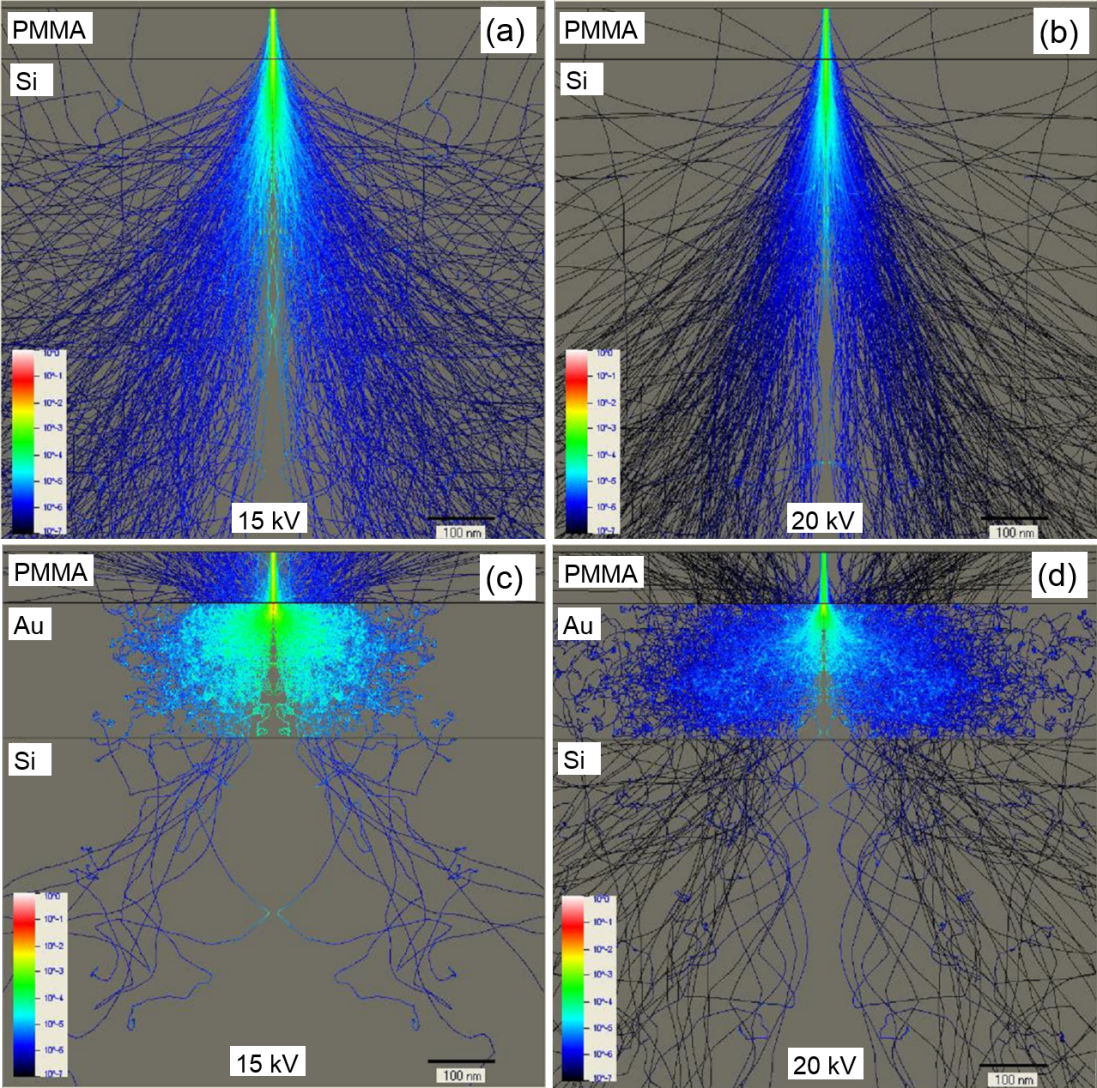
(a,b) Electron energy distribution in a 75 nm thick PMMA layer on a bulk Si substrate at 15 and 20 kV incident energies, respectively. (c, d) Energy distribution in a 75 nm thick PMMA layer on a Si substrate coated with a 200 nm Au film at 15 and 20 kV incident energies, respectively.

The dependence of the spot lithography resolution for a 75 nm thick PMMA layer on the acceleration voltage in the range of 10–20 kV can be illustrated with results from Monte Carlo simulations, as presented in Figure 4 and Figure 10. There are no significant differences in the energy distribution of electrons penetrating the resist for the Si substrate (Figure 4b and Figure 10a,b). However, for the Au substrate (in the case of a 10 kV acceleration voltage), the large angle backscattered and secondary electrons strongly contribute to the overexposure of the central pillar (Figure 4d). With an increase in the acceleration voltage, this effect diminishes due to the increase in the fraction of small angle backscattered electrons that expose the resist away from the central pillar (Figure 10c,d).

**Figure 11:**
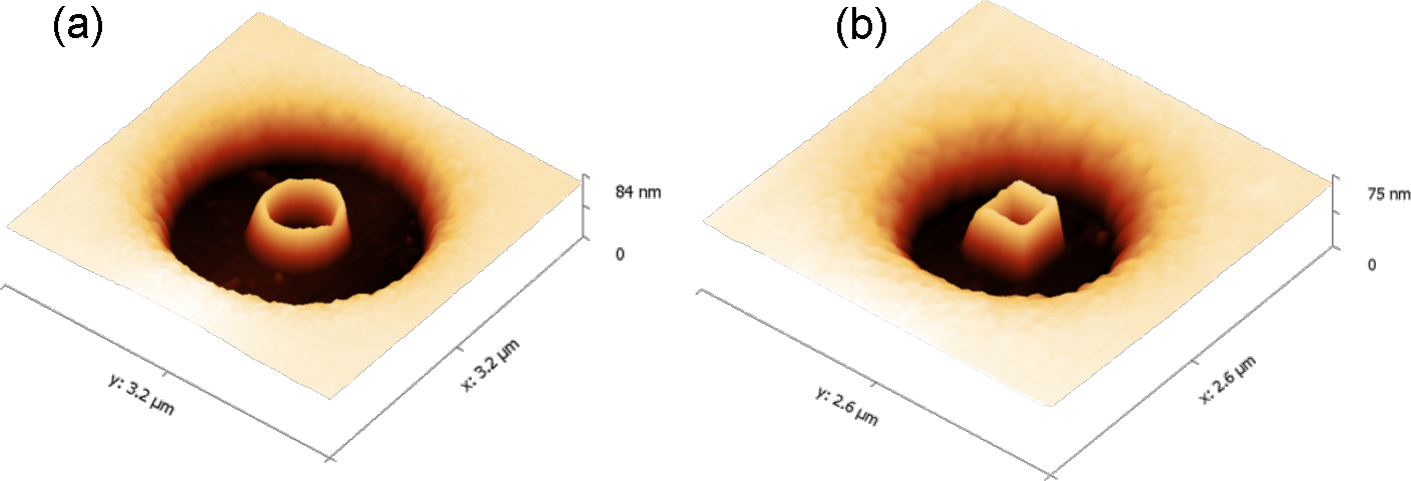
3D AFM images of polymer nanostructures fabricated on PMMA A2 resist with thickness of 75 nm on Si substrates at an acceleration voltage of 10 kV, exposure dose of 0.1 pC and *d*_s_ = 10 nm: (a) circular ring with a line width of 20 nm; (b) square ring with a line width of 22 nm.

Figure 11 provides three dimensional images of rings with round and square shapes made of carbonized resist. As can be seen, the nanostructures have flat outer and inner lateral sides with a thickness of about 20 nm, demonstrating the high resolution and spatial quality possible with this technique.

**Figure 12:**
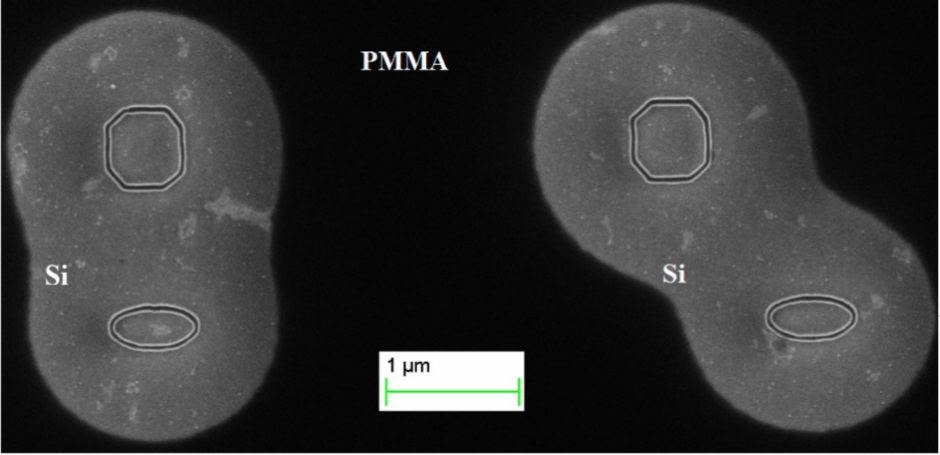
Demonstration of the proximity effect using nanostructures fabricated on a PMMA A2 resist with a thickness of 75 nm at a cathode acceleration voltage of 10 kV, exposure dose of 0.1 pC and *d*_s_ = 10 nm.

An example of the proximity effect is shown in Figure 12. If the nanostructures are close enough to one another, then surrounding areas, which behave as a positive-tone resist, are affected too. The black and grey contrast corresponds to PMMA and Si, respectively.

As has been shown above, the low energy single-spot electron-beam lithography method overcomes the disadvantages of conventional lithography on positive-tone resist. This method enables the formation of complex polymer nanostructures of any shape with a minimum line width of 10 nm not only on semiconductor substrates, but also on conductive surfaces such as a Au thin film. A similar sub-20 nm resolution is possibly using state-of-the-art, high-energy e-beam lithography systems with a beam acceleration of 50–100 kV on positive and negative resists [19]. In the single-spot nanolithography, there are no limitations in the use of thin metal films such as Au, Pt, Al, Cu, Ti or Cr. The minimum line width, given an electron beam diameter of 2 nm, is limited only by the length of the polymer chain (≈5 nm) and the thickness of the resist. We have empirically found that the thinner the resist, the higher the resolution of the single-spot lithography.

**Figure 13:**
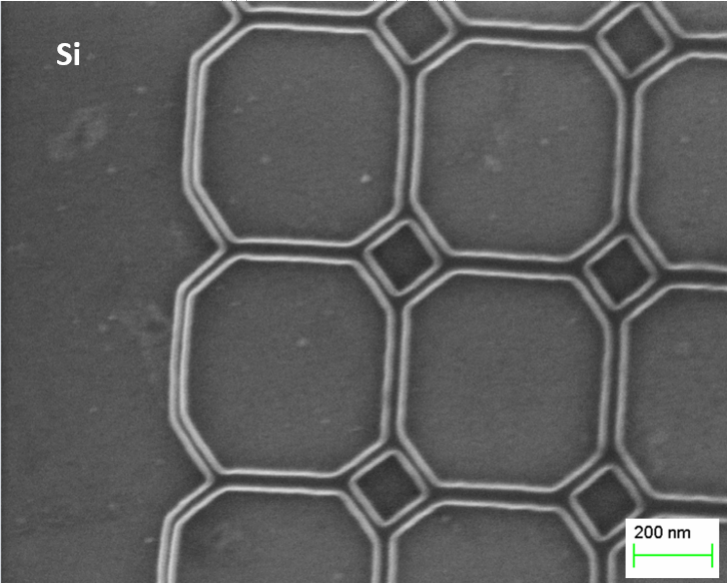
An example of a complex polymer pattern with a minimum line width of 15 nm fabricated at an acceleration voltage of 10 kV, exposure dose of 0.1 pC and *d*_s_ = 10 nm.

Another advantage is that the lines of patterned nanostructures are substantially defect-free, as shown in Figure 13. Our method enables the production of high-quality carbonized patterns of sub-20 nm resolution using the positive resist only. The transfer of patterns onto the substrates using wet chemical or plasma etching is possible due to the high chemical and mechanical resistance of the carbonized templates.

**Figure 14:**
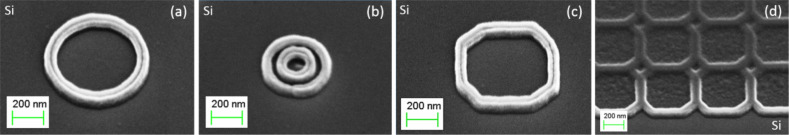
SEM images of Co nanostructures formed on the pre-patterned Si substrate: (a) circle, (b) double circle, (c) polygon, (d) complex polymer pattern shown in Figure 13.

An example of a practical application of the single-spot lithography method is shown in Figure 14. The patterned polymer nanostructures were sputtered with a magnetron in vacuum with a 20 nm-thick cobalt film. This resulted in 3D magnetic nanostructures with unusual spin configurations that potentially can be used in magnetic sensing technologies, memory, logic and biomedical applications [20]. Moreover, the single-spot nanolithography method is very promising for fabrication of high-quality, artificial, spin-ice lattices [21–22], magnonic [23] and photonic [24] crystals on large scale.

An important additional advantage of the proposed method is the fast patterning of a template as compared with the exposure time required for the reversed pattern on positive resist using conventional lithography techniques, where a sequential scan of the selected area is required. To fabricate a template in the form of an array of 25 × 25 (625 elements in total) equilateral triangles with a length of 210 nm and a line width of 40 nm, only 17.6 s are required. In contrast, the exposure of the same pattern using conventional lithography techniques on a positive resist requires 152 s, that is 8.6 times longer, but with reduced resolution.

## Conclusion

The fast and simple fabrication of polymer nanopatterns of various geometries with sub-10 nm resolution on semiconductor and metallized substrates using the single-spot nanofabrication was demonstrated at low-energy acceleration voltages. The resulting nanostructures have sharp edges and defect-free lines. Arrays of nanoelements or complex nanostructures can be easily scaled to large areas, with the distinct advantage of a reduced exposure time of up to ten times as compared to the conventional electron-beam lithography process on positive resist. The single-spot electron-beam lithography method enables the overexposure of selected areas of a positive resist with a dose higher then 0.1 pC and a distance between the spots of less than 30 nm. The resulting nanostructures are patterned in accordance with a digital pattern to produce the desired carbonized nanostructures. The high acid and plasma resistance as well as the presumably high mechanical durability of the carbonized nanostructures make them very attractive for the fabrication of hard nanoimprint lithography molds and etching masks, as well as for nanoelectronic and nanophotonic applications, MEMS and NEMS devices.

## Experimental

### Sample preparation

All the preparation procedures were conducted in a class 10,000 clean room. The PMMA A2 resist (MicroChem Corp., 950 K, 2 wt % in anisole) was spin-coated at different rates in range from 1000–4000 rpm for 1 min in order to produce the desired thickness on standard-cleaned undoped Si(111) substrates without and with a Au thin film of 200 nm thickness. The Au thin film was deposited by thermal evaporation of pure gold (99.99 atom %) from a Mo crucible at a base pressure of 10^−5^ Torr. The samples were baked on a hot plate at 180 °C for 90 s in order to harden the resist, to remove the residual solvent, and to raise the adhesion between PMMA and the substrate surface. Immediately after baking, the samples were exposed in E-Line EBL system (Raith, Germany). The spot size, cathode acceleration voltage, beam current and aperture size were 2 nm, 10 kV, 0.072 nA and 30 µm, respectively. Some experiments were performed at an acceleration voltage of 15 and 20 kV. The vacuum pressure in the work chamber was 1.5 × 10^−6^ Torr. Prior to exposure, a digital pattern of the desired nanostructures was prepared based on the single-spot overexposure. The samples were developed with 1:3 MIBK/IPA at 20 °C for 45 s, then rinsed in pure IPA and distilled water for 10 s, and finally dried with nitrogen gas.

### Characterization

The thickness and surface morphology of the PMMA and Au films were measured with an Ntegra Aura (NT-MDT, Russia) atomic force microscope. The evolution of the resist before and after development was studied with a Supra (Carl Zeiss, Germany) scanning electron microscope at an acceleration voltage of 10 kV. Monte Carlo simulations were performed with the NanoPECS software package.
